# *World of Change*: Reflections within an educational and health care perspective in a time with COVID-19

**DOI:** 10.1177/0020764020979025

**Published:** 2020-12-10

**Authors:** Janne Brammer Damsgaard, Ann Phoenix

**Affiliations:** 1 Department of Public Health, Section of Nursing, Aarhus University, Denmark; 2 Thomas Coram Research Unit, Institute of Education, University College London, UK

**Keywords:** Education, understanding, practical wisdom, change, resonance, sensory approach

## Abstract

**Background:**

Besides handling the physical impacts of COVID-19 there is more than ever a need to understand what can help when mental health is challenged. Within this context our practical wisdom – our ability to understand and recognise when ‘the other’, for example the patient, is feeling lonely or anxious is particularly important.

**Aim:**

This article aims to contribute to the understanding of how the competence of health professionals may be advanced by helping them develop the self-understanding essential to being wise practitioners.

**Method:**

The article is based on a discussion informed by reflections (written in Danish and translated into English) by Masters students (and registered nurses) participating in a university programme “Patient and user focused nursing”.

**Findings:**

The first part of the article considers a student nurse’s reflection on understanding herself and one of her patients. The second part considers reflections on the contemporary world of change from a student nurse trying to engage with a world she experiences as falling apart. The third part addresses the impact of resonant places and encounters on developing self/other understandings; encounters that may also be produced through songs and lyrics. The final part draws conclusions on how it is possible to reach understandings of oneself and others as student health practitioners in time of a pandemic.

**Conclusion:**

In the process of developing understanding and recognition, competence built on self-understanding is central for helping form health professionals into ‘wise practitioners’. It is concluded that the existential implications of the COVID-19 pandemic, paradoxically, may direct many people’s awareness to a more sensitive, resonant, attitude towards the other. For some, this may produce a more humanized world and perception of others. Within this perspective the arts may help us develop self-understanding and recognition of ‘the other’.

## Introduction

In the unprecedented period following lockdown in many countries in 2020, there are many concerns about mental health and how people can cope with the fears and stresses they have faced with the COVID-19. An international systematic review and meta-analysis found a high psychological burden among both medical staff and the general public with a great deal of psychological distress among patients (Luo et al., 2020). Luo et al., argue that there is an urgent need to understand what the massive existential change produced by COVID-19 does to us as humans.

Within this context our practical wisdom – our ability to understand and recognize when ‘the other’, for example the patient, is feeling lonely or anxious – is important. In this article, we define this ability as ‘practical wisdom’. To develop that ability, competences built on self-understanding are central and help to form health professionals into ‘wise practitioners’ (Damsgaard, 2019a, 2019b; Eriksen et al., 2014). In teaching health professionals, it is, therefore, important to focus on their values and competences in order to help develop their ability to engage with compassion in relations with ‘the other’. Health professionals have to develop the competence to sense the suffering of the other and make wise judgements and good decisions together with the patient. Such competences require a sensitive awareness which is most likely to be developed through close interactions with people (patients) involving processes of recognition (Damsgaard, 2020; Damsgaard et al., 2020; Rosa, 2019). According to the Danish philosopher and theologist K.E. Løgstrup one way in which such sensitivity can be reached is through engaging with artistic expression of all kinds. According to Løgstrup, art provides a special form of (re)cognition of the human being and the world, involving a sensory approach (Løgstrup, 1976).

This article contributes to the understanding of how the competences of health professionals may be advanced by helping them to develop the self-understanding essential to being wise practitioners. The discussion is informed by reflections (written in Danish and translated into English) by Masters students (and registered nurses) participating in a university program ‘Patient and user focused nursing.’ The discussion below also includes an invitation to reflect on the artistic expression in the lyrics from a song, selected because of its perspective on how schools, teachers and practitioners (if not alert) can drain curiosity and hinder openness and sensitivity to understanding ourselves and the other. The article is divided into four brief sections. The first part considers a student nurse’s reflection on understanding herself and one of her patients. The second part considers reflections on the contemporary world of change from a student nurse trying to engage with a world she experiences as falling apart. The third part addresses the impact of resonant places and encounters in developing self/other understandings; encounters that we argue may also be produced through songs and lyrics. The final part draws conclusions on how it is possible to reach understandings of oneself and others as student health practitioners in a time of pandemic. The article argues that the existential implications of the COVID-19 pandemic, paradoxically, may direct many people’s awareness to a more sensitive, resonant, attitude toward ‘the other’.

## Understanding oneself and the other

According to the philosopher Martha Nussbaum, being an educated person (and a competent and wise health professional) requires an urge to learn and understand other practices (Nussbaum, 1998). In the disjunctions and trauma produced by the COVID-19 pandemic, student nurses at Aarhus University reported that their observations of their patients were sharpened as they learned more than they had expected to about patients in isolation.

Student Nurse A expressed it this way:

‘At first the woman answered in a superficial way – being isolated was boring and the time felt endless. But after a while she opened up telling about a situation that was entirely new to her. Being afflicted with breathing difficulties and in need of oxygen was bad. But being alone and not having anyone to talk to was devastating. The woman lowered her eyes and cried as she told me about these feelings. The day after this she was discharged to self-isolation. No follow-up was arranged. When I was riding my bike home from work that afternoon, I thought a lot about this – no follow-up unless she contacted us. It is remarkable that so many people experience such (in a way) traumatizing events without anyone following up’.

According to the German philosopher Hans-Georg Gadamer, understanding is something that comes to us or happens to us, if we are *prepared* for it (Gadamer, 1960). This is what is meant when we say that understanding is always also *an act of understanding ourselves*. It is relational and related to our positioning. Understanding depends on ourselves and consequently also what we as human beings have brought with us, formed through upbringing, formative education and culture and the prejudices and expectations that we have adopted – for example from schools and practice. To put it briefly, an important part of understanding is produced from being open and receptive.

From Gadamer’s (1960) perspective, understanding is a *‘way of being*’ – which means that we use our own experience to be open, concrete and to show that one is present to the other (in the above example, the patient), keeping them in mind. This means that certain aspects of the world, such as the patients’ loneliness, can only be understood through understanding one’s own experiences. However, understanding is not a question of making the other person fit into our own pre-judgment, but instead continuously reflecting upon whether our own understanding is relevant to the other. Within the perspective of user’s (patients) and health professionals’ shared experiences (Mendel et al., 2010) collaborating and learning (Bower et al., 2015; Richards et al., 2016) there is a growing amount of initiatives such as for example Shared Decision Making (Klingaman et al., 2015, Lovell et al., 2018, Samalin et al., 2018) and Peer Support (Crisanti et al., 2019). The research findings challenge traditional views of professionalism and describe important implications for mental health services from the user’s perspective. The studies provide a clear indication of the importance of becoming an active participant in one’s own life, and the need for deeper understanding among the professionals in relation to user experiences and preferences for helpful care in periods of mental health crisis in order to optimize the care.

In the example above, the nurse was touched by the situation as she critically reflected on her practice. Her burgeoning understanding of the painful, poignant and isolating impact of COVID-19 clearly plays in her mind in that she reflects on it continuing to think about it as she rode home. Part of her rumination about this experience is her inability to provide what she understands that her patient needs; company and follow-up. However, her understanding is necessarily limited in that her empathy is not equivalent to knowing, for example, the pain her patient felt. Understanding the other is not a method that allows us to become acquainted with the other person’s horizon but an ability wisely to engage with compassion in relation with the other *recognizing* the existential and mental impacts of COVID-19 pandemic.

## World of Change

Student nurse B began an essay with this incisive quotation and reflection:

‘It is difficult not to fall apart when things around you crumble’. This is what the Danish poet Tove Ditlevsen wrote in her poem ‘The Street of Childhood’ published in 1943 during World War Two (Ditlevsen, 1943). Today nearly 80 years later things are again dramatically changing. ‘This time a virus, a pandemic, has inflicted the world. We call it COVID-19 crisis’.

This is an evocative description of what occurs when we are ‘exposed’ to overwhelming life changes – when our lifeworld loses solid ground – when we are not in balance with our past, our present and our expectations for the future. Indeed, the COVID-19 virus is an overwhelming occurrence causing worldwide feelings of loss of solid ground. This is perhaps indicated by the student nurse’s use of metaphor and poetic expression to try to gain an understanding of the impact of the COVID-19 pandemic and to express her/his feelings about it.

According to the German sociologist Hartmut Rosa (Rosa, 2014), the continuous acceleration of social change, puts us under increasing pressure to keep up with technological, economic and social developments, causing social alienation, increasing burnout and depression. Rosa developed his theory of social acceleration before the COVID-19 pandemic. His theory is even more pertinent at a time when the pandemic means that our institutions and practices are marked by the ‘shrinking of the present’, a decrease in time during which expectations based on past experience reliably match the future. This makes our relationships with each other and to the world fluid and problematic. Melancholia and depression are intensified when the changes in our lives (in the social world) are no longer experienced as ‘elements in a meaningful and directed chain of developments’, that is, as elements of ‘progress’, but as directionless, ‘frantic’ change (Rosa, 2014). When we as human beings (patients) feel that we have lost control of our direction in life it can affect our way of understanding ourselves and our connectedness, hope, identity, meaning and empowerment. This is the situation reflected on by the student nurse cited at the start of this section.

Yet, from Rosa’s viewpoint, positive (dynamic) change is possible when episodes of change are perceived to add up to a (narrative) story of growth or progress. This notion of growth and progress is crucial for health professionals and students. Although paradoxical, many commentators are now arguing that, if we are able to understand this traumatic period and seize the moment, the COVID-19 crisis may function globally as an eyeopener to the need for a more humanized world. This humanized world would be one marked by social justice and produce an awareness to the importance of a sensitive, resonant, attitude toward the other. As Simon Mair (2020) suggests ‘A key task for us all is demanding that emerging social forms come from an ethic that values care, life, and democracy. The central political task in this time of crisis is living and (virtually) organizing around those values.’ The section below discusses this resonance by considering the place of music.

## Resonant places and encounters

For many people, education is a site important to the relationships people develop with the world. It is in education that we are most likely to have to grapple with the ‘stuff of the world’ by reflecting on it, actively distancing ourselves from it, and adaptively transforming it, as we begin to formulate and articulate our moral roadmap. Within this context, education becomes a critical, constitutive ground for the development or closure/obstruction of axes of resonance. Our experiences in and around the classroom (including in clinical educational settings) determine both what sensitivities or insensitivities to resonance we will have access to in dealing with the potential meanings we encounter in the world. At its heart, the educational process consists in learning how we relate to or (have to) position ourselves in relation to the various spheres of action and life. From this perspective the relation and resonance can only be established when we discover that we can achieve or move something, that is, that the music or the poem ‘responds’ to us (Damsgaard, 2019b; Rosa, 2019).

The group Supertramp has dealt with the school experience. In their music and texts, school functions as institutions which unfortunately often seem to transform our (resonant) relationship to the world into a ‘mute’ relationship that ultimately confronts life with cynicism.

‘The Logical Song’ expresses this simply but vividly:

When I was young

It seemed that life was so wonderful

A miracle, oh it was beautiful, magical

And all the birds in the trees

Well they’d be singing so happily

Joyfully, playfully watching me

But then they send me away

To teach me how to be sensible

Logical, responsible, practical

And then they showed me a world

Where I could be so dependable

Clinical, intellectual, cynical

There are times when all the world’s asleep

The questions run too deep for such a simple man

Won’t you please tell me what we’ve learned?

I know it sounds absurd but please tell me who I am

To Rosa it is clear that school not only opens or closes individual axes of resonance, but also forms the quality of our relationship to the world as a whole. As a teacher (and a health professional) awareness of this perspective is crucial. It is important constantly to reflect on whether we create ‘mute’ relationships, or whether we as teachers and professionals will be able to reach our students and patients.

Student nurse C working with COVID-19 patients expressed a pivotal moment in sharing this reflection:

‘Especially after his wife had passed away, he had felt lonely and had become more dependent on his relatives whom he had not seen for several weeks caused by fear of COVID-19 virus. Because of his dementia he had difficulties separating the days from each other, remembering details. “But I still have my feelings – and I miss my family so much that it hurts” he told me. That hit me right in the heart and I should really pull myself together to keep the two-metre safety zone. This made me reflect on loneliness and gratitude for having someone to talk to’.

This written reflection is entirely opposite to the ‘clinical, intellectual, cynical’ impact of education presented in Supertramp’s song. While certainly not about the joy Supertramp identify in the pre-educated state, it shows a raw openness to feeling in ‘that hit me right in the heart’. Regardless of what this student nurse is learning in the classroom, the hospital room constitutes a place of resonance. For them, the COVID-19 crisis is functioning as a humanizing eyeopener to the need for a more humane world. Such experiences of sensitivity and connectedness are important to highlight and begin to learn from, in grasping what a resonant encounter is about at this time of unprecedented change.

## Using the arts to reach oneself and the other

The Supertramp song above provides one way into drawing on insights about the place of education for developing resonance that can be applied to working with patients experiencing the most psychologically painful aspects of living with COVID-19. If we want to open up to existential aspects of human existence, we (teachers and professionals) may find help in all varieties of art. Art functions as a catalyst and thereby is a means of gaining insight into reality, which may otherwise be neglected or put aside. Reading, listening to music or going to the theatre, the opera, the ballet, to the cinema and to concerts as well as looking at art in city spaces and museums – or any other sort of creative activity may all help. This is because perception, understanding and resonance work together in art. In Løgstrup’s words, ‘the artist bears in mind the perceptive element per se’ (Løgstrup, 1976). Art entails cognition, and art provides a particular form of (re)cognition of the human being and of the world. It is a means to gain insight which may otherwise be neglected or ignored.

Art is also different from the recognition that we find in our everyday life and in science. In the everyday we often find ourselves immediately in the world, actively doing one thing or another in ways that neglect the sensory approach to the world. If we can manage to articulate it, art contains a certain recognition (resonance), which may in turn be of greater importance than the recognition we gain from theories, information, standards etc. (Løgstrup, 1976). A growing amount of research documents the evidence of the role of the arts in improving health and wellbeing (Jensen, 2017). A literature review by Anita Jensen (2017) documents that art, culture and creative activities can enhance mental health. The effects are reported as feelings of wellbeing, being a part of a group, building new relations, participating in meaningful activities, improving self-esteem, enhancing motivation and aspiration, creating connection between body and mind, decreasing depressive feelings, enhancing relaxation, enhancing self-confidence, enhancing hope and developing new coping strategies (Jensen, 2017). These results are supported in a scoping review by WHO stating that there is ‘. . . evidence of the contribution of the arts to the promotion of good health and the prevention of a range of mental and physical health conditions, as well as the treatment or management of acute and chronic conditions arising across the life-course’ (World Health Organization, 2019). The report also finds that implementing art in clinical practice may help in providing multisectoral, holistic and integrated person-centered care, addressing complex challenges for which there are no current health care solutions (World Health Organization, 2019).

Through art we may train our senses, for example, looking, hearing, touching, responding – to the impressions that we receive. In this process we intensify and clarify our impressions, because we establish experience through our sensory understanding. Through recognition in artistic depictions we can learn the art of interpretation and let it grow and take form. Through the transformational power of art we may, therefore, find ourselves and the other.

## Conclusion

To conclude our practical wisdom – our ability to understand and recognize when ‘the other’, for example the patient, is feeling lonely or anxious is crucial. In that process, competences built on self-understanding are central for helping to form health professionals into wise practitioners.

In teaching health professionals, it is, therefore, important to focus on their values and competences in order to help develop their ability to engage with compassion in relations with the other. However, when we as human beings feel that we have lost control of our direction in life it can affect our way of understanding ourselves and our connectedness, hope, identity, meaning and empowerment. Within this context it is argued that the existential implications of the COVID-19 pandemic, paradoxically, may direct many people’s awareness to a more sensitive, resonant, attitude toward the other. If we are able to understand this traumatic moment, the COVID-19 crisis may function globally as an eyeopener to the need for a more humanized world, marked by social justice and produce an awareness to the importance of a sensitive, resonant, attitude toward the other.

Within this light it is important constantly to reflect on whether we create ‘mute’ relationships, or whether we as teachers and health professionals are able to reach our students and patients. It is suggested that if we want to open up to existential aspects of human existence, we (teachers and professionals) may find help in art of all kinds. Art functions as a catalyst and thereby is a means to gain insight in reality, which may otherwise be neglected or put aside. Perception, understanding and resonance work together in art. On that basis it is argued that art provides a special form of (re)cognition of the human being and the world involving a sensory approach toward the other enhancing mental health.
